# Extended Interfacial Charge Transference in CoFe_2_O_4_/WO_3_ Nanocomposites for the Photocatalytic Degradation of Tetracycline Antibiotics

**DOI:** 10.3390/molecules29194561

**Published:** 2024-09-25

**Authors:** Suiying Dong, Jiafu Dai, Ying Yang, Amir Zada, Kezhen Qi

**Affiliations:** 1 Yunnan Provincial Key Laboratory of Entomological Biopharmaceutical R&D, College of Pharmacy, Dali University, Dali 671000, China; 15198908547@163.com (S.D.);; 2 Asset and Laboratory Management Division, Dali University, Dali 671000, China; yangying@dali.edu.cn; 3 Department of Chemistry, Abdul Wali Khan University, Mardan 23200, Pakistan; 4 UNESCO-UNISA Africa Chair in Nanosciences and Nanotechnology, College of Graduate Studies, University of South Africa, Muckleneuk Ridge, P.O. Box 392, Pretoria 0002, South Africa

**Keywords:** WO_3_, CoFe_2_O_4_/WO_3_, loading, tetracycline, photocatalytic degradation

## Abstract

The large-scale utilization of antibiotics has opened a separate chapter of pollution with the generation of reactive drug-resistant bacteria. To deal with this, in this work, different mass ratios of CoFe_2_O_4_/WO_3_ nanocomposites were prepared following an in situ growth method using the precursors of WO_3_ and CoFe_2_O_4_. The structure, morphology, and optical properties of the nanocomposite photocatalysts were scrutinized by X-ray diffraction (XRD), UV-visible diffuse reflectance spectra (UV-Vis DRS), photoluminescence spectrum (PL), etc. The experimental data signified that the loading of CoFe_2_O_4_ obviously changed the optical properties of WO_3_. The photocatalytic performance of CoFe_2_O_4_/WO_3_ composites was investigated by considering tetracycline as a potential pollutant. The outcome of the analyzed data exposed that the CoFe_2_O_4_/WO_3_ composite with a mass ratio of 5% had the best degradation performance for tetracycline eradication under the solar light, and a degradation efficiency of 77% was achieved in 20 min. The monitored degradation efficiency of the optimized photocatalyst was 45% higher compared with the degradation efficiency of 32% for pure WO_3_. Capturing experiments and tests revealed that hydroxyl radical (·OH) and hole (h^+^) were the primary eradicators of the target pollutant. This study demonstrates that a proper mass of CoFe_2_O_4_ can significantly push WO_3_ for enhanced eradication of waterborne pollutants.

## 1. Introduction

Antibiotics are a class of secondary metabolites, also known as antimicrobial materials, produced by microorganisms (bacteria, fungi, and actinomycetes) or synthesized synthetically. They are widely used in medicines, aquaculture, animal husbandry, and other fields to treat fatal diseases caused by microbial infections [1,2]. The widespread use of antibiotics, although providing benefits, has resulted in the introduction of antibiotic accumulation in water bodies, causing water pollution that largely affects the stability of ecosystems [3]. It has caused serious environmental issues, and antibiotics pose ecological risks by affecting microbial diversity and plant growth and development, with enormous effects on human health [4]. In recent years, the potential risk of a new tetracycline antibiotic to the environment and human health has attracted widespread attention as it is a typical broad-spectrum antibiotic and widely used in health care centers, aquaculture, and animal husbandry [5,6]. Therefore, finding efficient methods to control the mass of tetracycline in water bodies has become a current research hotspot, which has far-reaching practical impact in the protection of ecological environments.

Photocatalytic degradation of tetracycline has many advantages as it is a potential method to solve environmental problems. This method uses light energy to decompose organic pollutants without additional chemical reagents and is environmentally friendly [7]. Photocatalytic technology, as an advanced oxidation method, is based on the principle of solar energy conversion into chemical energy with the aid of oxidation realized in the presence of oxygen reactive species such as ·OH, ·O_2_^−^, and ^1^O_2_ produced during the photocatalytic process. The role of the photocatalyst in the reaction process is to direct the reactants (organic pollutants) to undergo a chain oxidation-reduction reaction towards the degradation of pollutants into CO_2_, H_2_O, and some inorganic ions, while ensuring that it itself remains unchanged before and after the reaction [8,9,10]. Under light conditions, excitation of the photocatalyst generates electrons (e^−^) in the conduction band and holes (h^+^) in the valence band [11]. The excited e^−^-h^+^ combine with O_2_ and H_2_O to form free radicals to completely oxidize and degrade the pollutants [12,13,14]. It should be noted that the photogenerated carriers formed by the excitation of the material should be properly separated under the action of an internal electric field for migration to the catalyst surface. The produced electrons and holes chemically react with O_2_ and H_2_O/OH^−^ to give birth to superoxide radical (·O_2_^−^) and ·OH/H_2_O_2_ free radicals, which effectively remove all the toxic materials present in water bodies [15,16]. The recombination of e^−^ and h^+^ in this process leads to a decrease in the active electron and hole lifetime, which reduces the degradation efficiency of catalysts [17,18,19,20,21].

The forbidden bandwidth of WO_3_ is adjustable in the range of 2.5–3.5 eV, while the potential of its valence band is about 3.2 V. This potential endows a strong oxidation ability to effectively remove many toxic materials [22,23,24]. However, WO_3_ still has the disadvantages of high electron–hole pair recombination rates and narrow light absorption range, which greatly restrict its practical application [25,26,27]. Cobalt ferrate (CoFe_2_O_4_) as a typical spinel-type ferrite has widely been used in photocatalysis owing to its permanent magnetism, saturation magnetization strength, magnetostriction coefficient, and relatively large magnetic crystal anisotropy constant. Its physico-chemical stability is relatively good, and it has been widely applied in capacitor energy storage, advanced oxidation, biomedicine, and many other aspects [28,29,30]. CoFe_2_O_4_ has a band gap around 1~3 eV, and its ferromagnetism boosts rapid separation from liquid–solid systems under external magnetic fields [31]. CoFe_2_O_4_ nanomaterials can fully utilize visible light to achieve high light utilization. Therefore, loading a certain amount of CoFe_2_O_4_ on the WO_3_ surface will greatly improve its photocatalytic performance.

In this paper, WO_3_ photocatalyst was prepared by a hydrothermal method and loaded with different masses of CoFe_2_O_4_ to obtain CoFe_2_O_4_/WO_3_ composite photocatalysts. The photocatalysts were checked under solar light to degrade tetracycline antibiotic as a potential pollutant in drinking water bodies.

## 2. Results and Discussion

### 2.1. Phase Analysis

**Figure 1:**
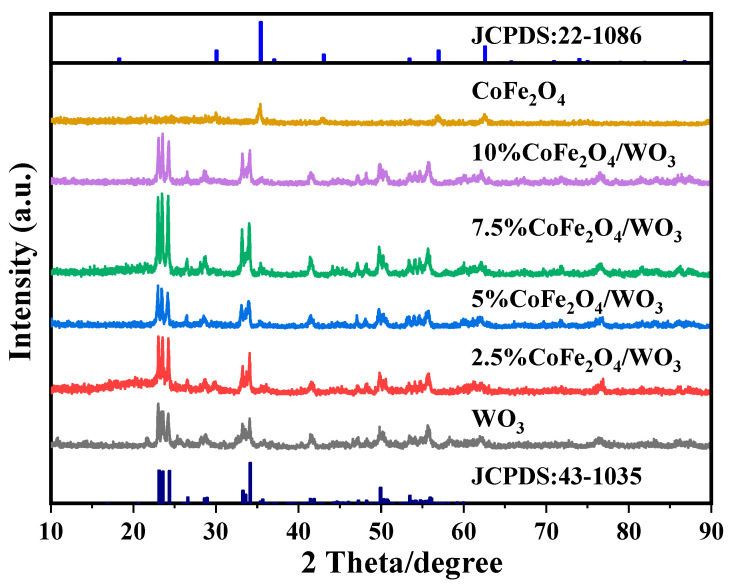
XRD patterns of WO_3_, CoFe_2_O_4_, and CoFe_2_O_4_/WO_3_ samples.

The XRD data in Figure 1 show the characteristic diffraction peaks of WO_3_ at 2θ values of 22.98°, 23.62, 24.24°, 34.08°, and 49.82°, which correspond to the (002), (020), (200), (202), and (140) crystal planes in the standard card JCPDS:43-1035. The results indicate monoclinic crystalinity of WO_3_ [32]. The introduction of CoFe_2_O_4_ basically does not affect the position of the diffraction peaks of WO_3_, proving that it does not change the crystalline structure of WO_3_ [33]. Individual diffraction peaks of CoFe_2_O_4_ are masked by the diffraction peaks of WO_3_. At the same time, no other obvious diffraction peaks are detected, showing that the samples are highly crystalline.

### 2.2. Morphological Analysis

**Figure 2:**
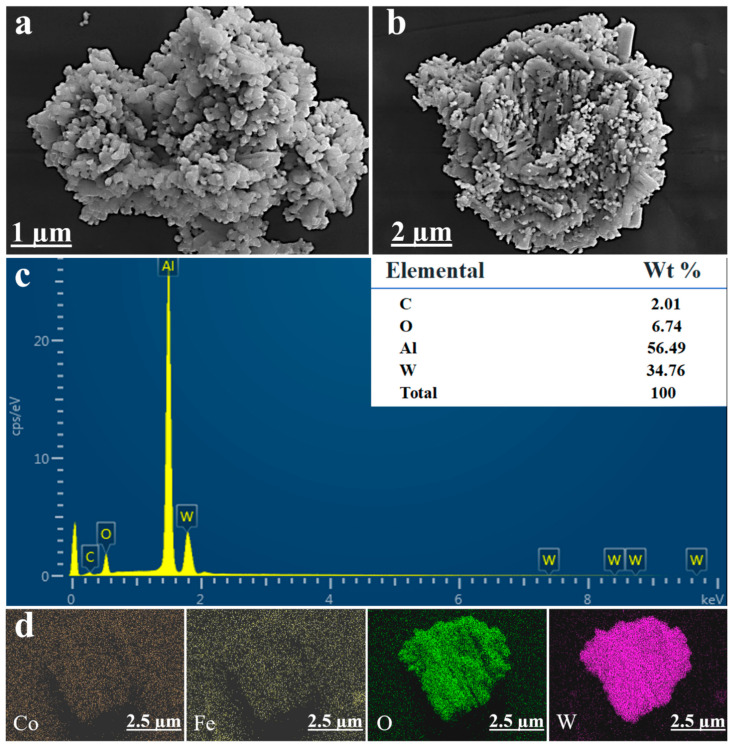
(**a**,**b**) SEM images, (**c**) EDS energy spectra, and (**d**) elemental mapping analysis of Co, Fe, O, and W for the sample of 5%CoFe_2_O_4_/WO_3_.

Figure 2a,b show the SEM images of 5%CoFe_2_O_4_/WO_3_. It can be understood that the surface of photocatalysts is rough and is made of countless nanosheets and fine particles with visible voids, which are conducive to photocatalysis. The nanosheet surface of WO_3_ is tightly attached and bonded with CoFe_2_O_4_, which proves that CoFe_2_O_4_/WO_3_ composites are successfully prepared. Figure 2c demonstrates that all the constituent elements are present in the composite, as shown in the EDS energy spectrum. However, the elements of CoFe_2_O_4_ are not detected, which may be due to its low content. The presence of the C element is attributed to some low boiling point volatile organic compounds in the air adsorbed on the sample surface, and the Al element is introduced from the test carrier. No other impurity signals are found, indicating that the composite material does not contain impurities. Figure 2d shows that all four elements, Co, Fe, O, and W, are present in the CoFe_2_O_4_/WO_3_ composite and indirectly indicates that the absence of CoFe_2_O_4_ in the EDS spectra is due to its low content.

### 2.3. Energy Band Structure

**Figure 3:**
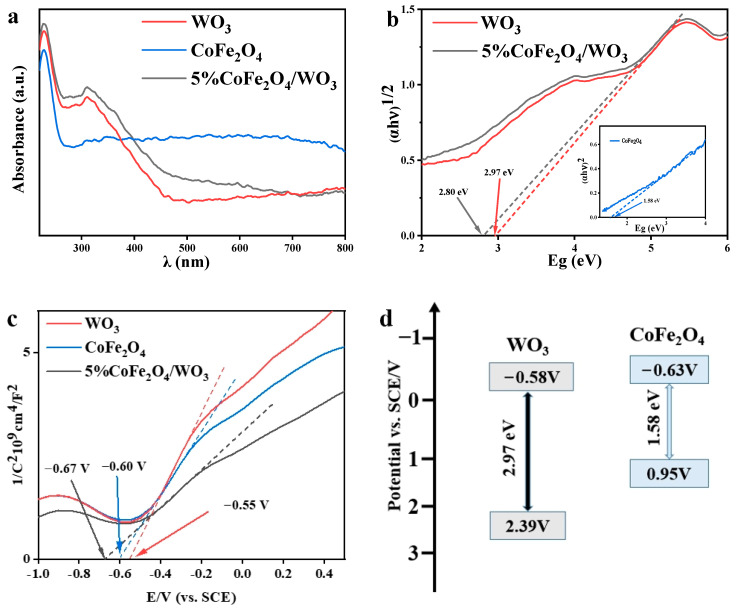
(**a**) UV-visible diffuse reflectance spectra; (**b**) forbidden bandwidth; (**c**) plot of flat-band potential; (**d**) energy band structure of the samples.

The optical properties of WO_3_, CoFe_2_O_4_, and 5%CoFe_2_O_4_/WO_3_ were characterized by UV-Vis diffuse reflectance, as presented in Figure 3a,b. WO_3_ and 5%CoFe_2_O_4_/WO_3_ samples absorb UV-visible light in the range of 220–800 nm. The absorption intensity of 5%CoFe_2_O_4_/WO_3_ is higher than that of WO_3_ in the wavelength range of 220–690 nm, and the absorption edges of samples appear to be red shifted, indicating that the absorbance capacity of 5%CoFe_2_O_4_/WO_3_ is better than that of WO_3_, and the absorption performance of the composites in the UV-visible region has been improved to some extent [34].

The Tauc-plot equation is as follows [35,36]:

αhν = A(hν − E_g_)^n/2^ (1)

E_g_ = hν (2)

Here, α, A, hν, and E_g_ are the absorption coefficient, constant, photon energy, and band gap energy, respectively. The value of n is related to the type of semiconductor when the material is a direct transition semiconductor, the value of “n” is 1. When the material is an indirect transition semiconductor, the value of “n” is 4. According to the above equation, a plot of “hν” corresponding to “(αhν)^2/n^” was drawn. The tangents intersect the *x*-axis at 2.97 eV, 2.80 eV, and 1.58 eV, corresponding to the forbidden band widths (E_g_) of WO_3_, 5%CoFe_2_O_4_/WO_3_, and CoFe_2_O_4_ [37,38]. From the experimental data, the band gap energy of the prepared 5%CoFe_2_O_4_/WO_3_ is relatively low compared with WO_3_, as evidenced by the fact that 5%CoFe_2_O_4_/WO_3_ is more easily excited by visible light with a broadened spectral response range. Thus, the introduction of CoFe_2_O_4_ leads to the reduction of the band gap energy of WO_3_, and stronger light absorption may be one of the reasons for its higher photocatalytic efficiency.

To measure the flat-band potentials, the impedance–potential method was used for WO_3_, 5%CoFe_2_O_4_/WO_3_, and CoFe_2_O_4_. The measured data obtained were inverted by the Mott-Schottky theory. The obtained Mott–Schottky curves for the catalysts of WO_3_, 5%CoFe_2_O_4_/WO_3_, and CoFe_2_O_4_ are provided in Figure 3c. The slopes of the curves are all greater than 0, signifying that the samples are all n-type semiconductors. The intercepts of the tangent lines on the *x*-axis of the Mott–Schottky curves for the catalysts of WO_3_, 5%CoFe_2_O_4_/WO_3_, and CoFe_2_O_4_ are −0.55 V, −0.67 V, and −0.60 V, respectively (vs. SCE). The value of flat-band potentials (E_fb_) of the material was calculated according to Equation (3) [39]:

(3) $$E_{0}=E_{fb}+\frac{RT}{F}$$

Here, E_0_ is the intercept of the tangent line of the Mott–Schottky curve, R is the gas constant, T is the temperature, and F is the Faraday’s constant. The calculated E_fb_ of WO_3_, 5%CoFe_2_O_4_/WO_3_, and CoFe_2_O_4_ are −0.58 V, −0.70 V, and −0.63 V, respectively.

Normally, it is generally accepted that the value of the flat-band potential of an n-type semiconductor is approximately equal to the value of the conduction-band potential [40,41]. The valence band potential of the sample was calculated according to Equation (4):

E_CB_ = E_VB_ − E_g_ (4)

Here, E_CB_, E_VB_, and E_g_ represent the CB potential, VB potential, and band gap energy, respectively. As demonstrated in Figure 3b, the E_g_ of WO_3_, 5%CoFe_2_O_4_/WO_3_, and CoFe_2_O_4_ are 2.97 eV, 2.80 eV, and 1.58 eV, respectively. From the values of CB potential and E_g_, the VB potentials of WO_3_, 5%CoFe_2_O_4_/WO_3_, and CoFe_2_O_4_ are 2.39 V, 2.10 V, and 0.95 V, respectively. Based on the above experimental results, the visualized energy band structure is shown in Figure 3d.

### 2.4. BET Surface Area Analysis

The specific surface areas, pore diameters, and pore volumes of CoFe_2_O_4_, WO_3_, and 5%CoFe_2_O_4_/WO_3_ are displayed in Table 1. The specific surface area of 5%CoFe_2_O_4_/WO_3_ is smaller than WO_3_, which may be attributed to the arrival of CoFe_2_O_4_ in the nanocomposite. In general, the smaller the specific surface area of the catalyst, the fewer the active sites. Accordingly, the contact between the catalyst and the pollutant reduces, which is not conducive to the improvement of the degradation efficiency of the catalyst [42,43].

**Figure 4:**
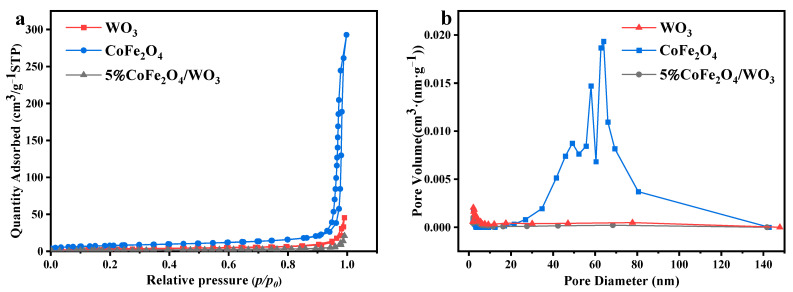
(**a**) Nitrogen adsorption and desorption diagrams of CoFe_2_O_4_, WO_3_, and 5%CoFe_2_O_4_/WO_3_ samples; (**b**) pore size distributions.

The nitrogen adsorption and desorption diagrams of CoFe_2_O_4_, WO_3_, and 5%CoFe_2_O_4_/WO_3_ are shown in Figure 4a. WO_3_ and 5%CoFe_2_O_4_/WO_3_ have similar shapes; both have desorption hysteresis loops in the range of P/P_0_ = 0.2~1.0, which suggests that the materials have mesoporous structures [44]. The adsorption capacity of the three materials increases with an increase in P/P_0_, which is relatively low in a certain range. When P/P_0_ = 0.9, the adsorption capacity has a large increase. Overall, the adsorption capacity of the three materials (CoFe_2_O_4_ > WO_3_ > 5%CoFe_2_O_4_/WO_3_) is in accordance with the results of the specific surface area test. Figure 4b shows that the pore volume is reduced significantly after loading CoFe_2_O_4_, indicating that the reduction of composite pore volume is mainly attributed to CoFe_2_O_4_. The decrease in specific surface area and pore volume may be due to the close interfacial contact between CoFe_2_O_4_ and WO_3_ [45].

### 2.5. Photodegradation Study

**Figure 5:**
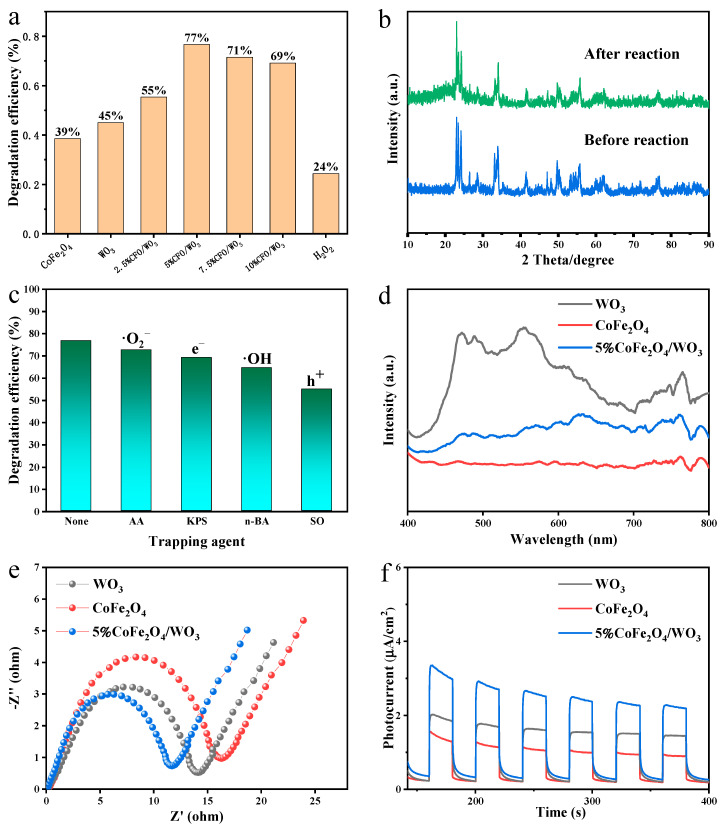
(**a**) Photocatalytic degradation performance of tetracycline for samples; (**b**) XRD patterns of 5%CoFe_2_O_4_/WO_3_ before and after reaction; (**c**) Effect of different capture agents on the performance of photocatalytic degradation of tetracycline with 5%CoFe_2_O_4_/WO_3_; (**d**) fluorescence spectroscopy; (**e**) alternating current impedance spectra; and (**f**) photocurrent response.

The dark adsorption equilibrium time for the photocatalytic degradation experiments was 30 min, and the degradation experiments were conducted after the adsorption equilibrium. Experiments were steered to investigate the effect of different CoFe_2_O_4_ loadings on the photocatalytic performance, and the results are displayed in Figure 5a. CoFe_2_O_4_/WO_3_ with CoFe_2_O_4_ percentage content of 2.5%, 5%, 7.5%, and 10% were synthesized for the degradation of tetracycline. The experimental results show that the introduction of CoFe_2_O_4_ can significantly improve the photocatalytic degradation activity of TC by WO_3_, and the best degradation performance is achieved when the compound ratio of CoFe_2_O_4_ is 5%. After that, the degradation efficiency of tetracycline gradually decreases with an increase in the loading ratio, which may be attributed to the excessive occupation of the active sites on the catalyst surface by the loaded CoFe_2_O_4_. The experimental results also show that hydrogen peroxide alone does not have a better degradation efficiency for TC.

Regarding the stability of the materials, we compared the XRD patterns of the optimized photocatalyst with the best catalytic performance before and after the degradation experiments to analyze whether its crystalline phase was changed, as shown in Figure 5b. The positions and intensities of the characteristic peaks of the XRD patterns before and after the reaction are almost unchanged, indicating that the composites are stable.

Potassium persulfate (KPS), ascorbic acid (AA), n-butanol (n-BA), and sodium oxalate (SO) were added to the solution as trapping agents for e^−^, ·O_2_^−^, ·OH, and h^+^ to validate the experimental mechanism, while maintaining the same conditions for the photodegradation experiments [46,47]. At the end of dark adsorption, the samples were added with 1 mL of capturing agent and irradiated under solar light for 100 min. The results in Figure 5c show that the degradation efficiency of the catalyst decreases to some extent after the addition of the capture agent. The experimental results show that e^−^, ·O_2_^−^, ·OH, and h^+^ all play certain roles in the degradation of tetracycline, while ·OH and h^+^ are the main active substances that play a role in the degradation.

At the maximum excitation wavelength, fluorescence spectroscopy was measured, as shown in Figure 5d. It is well known that a higher fluorescence intensity represents a higher rate of electron–hole combination [48]. Comparing the fluorescence intensity of 5%CoFe_2_O_4_/WO_3_ and WO_3_, the fluorescence intensity of 5%CoFe_2_O_4_/WO_3_ < WO_3_, which indicates that the introduction of CoFe_2_O_4_ inhibits the recombination of e^−^ and h^+^ to a certain extent. As a result, the lifetimes of the photogenerated carriers of 5%CoFe_2_O_4_/WO_3_ are prolonged, which improves their photocatalytic performance.

To investigate the effect of CoFe_2_O_4_ loading on the charge transfer efficiency of the catalysts, electrochemical impedance spectroscopy (EIS) was measured in a 0.1 mol/L KCl solution containing 5 mM K_3_[Fe(CN)_6_]/K_4_[Fe(CN)_6_]. As shown in Figure 5e, it can be seen that 5%CoFe_2_O_4_/WO_3_ has a lower arc radius than WO_3_ in the high-frequency region of the EIS Nyquist plot, so 5%CoFe_2_O_4_/WO_3_ has a higher carrier transfer efficiency [49].

To further understand the effective separation efficiency of the photogenerated electron–hole pairs of the samples, we performed photocurrent experiments in NaSO_4_ (0.1 mol/L) electrolyte solution to detect the intensity of the photocurrent generated by the materials. The interval between the light switched on and switched off was 20 s, and the measurements were completed in 300 s. Largely, the stronger the photocurrent, the more efficient the separation of electrons and holes, and the better the performance of the photocatalyst [50,51]. From Figure 5f, it can be seen that the photocurrent response of 5%CoFe_2_O_4_/WO_3_ is stronger than that of WO_3_ and CoFe_2_O_4_ throughout the testing time. This signifies that the photogenerated electron–hole recombination rate is reduced after loading CoFe_2_O_4_ compared to that of the pure WO_3_, which is conducive to the enhancement of the photoelectrochemical performance of WO_3_.

### 2.6. Photocatalytic Mechanism

**Figure 6:**
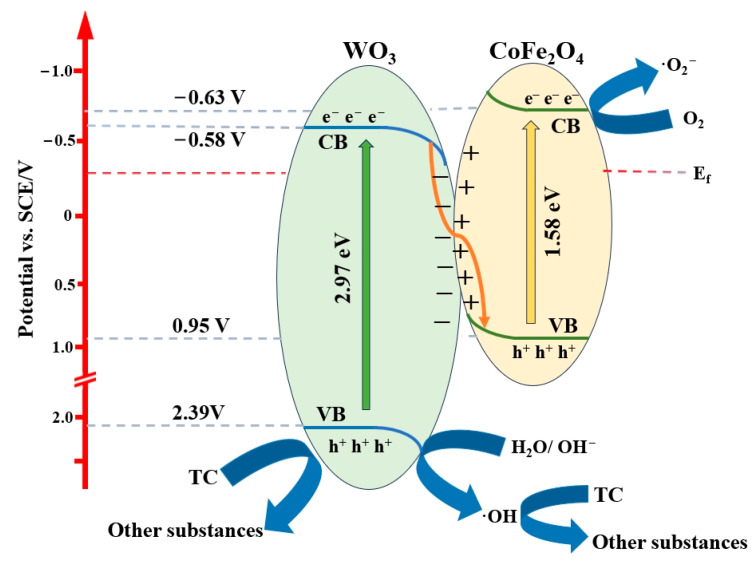
Reaction mechanism of the photocatalytic degradation of tetracycline by CoFe_2_O_4_/WO_3_.

Based on the results of the free radical trapping experiments, energy band structure analysis, and photocatalytic degradation efficiency, we propose a possible mechanism for the photocatalytic degradation of tetracycline. On one hand, compared with the CoFe_2_O_4_ pure phase material, the photocatalytic degradation efficiency is significantly increased after composite with WO_3_. As shown in Figure 5a, the degradation efficiency increases from 39% to 77%, demonstrating an increase of 38%. Combined with the results of the capture experiments and energy band structure analysis, it suggests that the oxidation reaction may be dominated by holes in VB with a higher oxidation potential. In other words, oxidation occurs in the VB of WO_3_ with a more positive potential. On the other hand, the photocatalytic degradation efficiency of the composites is also significantly improved with the introduction of 5%CoFe_2_O_4_ into the materials compared with WO_3_. As shown in Figure 5a, the degradation efficiency increases from 45% to 77%, showing an increase of 32%. Also, combining the results of the trapping experiments and energy band structure analysis, we believe that the photogenerated hole and electron pairs of the composites are separated more efficiently, which results in more photogenerated holes and electrons participating in the degradation reaction. Meanwhile, this conjecture is also verified by the results of photoluminescence, photocurrent, and AC impedance experiments. Coincidentally, this scenario fits well with the S-scheme heterojunction building theory. When irradiated by solar light, CoFe_2_O_4_ and WO_3_ materials are excited when the energy of the incident light is larger than their band gap energies. As a result, the electrons in VB jump to the CB, generating high electron density in the CB and photogenerated h^+^ in VB of the two components [52]. As shown in Figure 6, when the two semiconductor materials are compounded together, the contact surface and difference in the Fermi energy levels drive the electron transfer from the CoFe_2_O_4_ surface with a higher reduction potential to the WO_3_ surface, which leads to the formation of a built-in electric field. The direction of the electric field is from the reduced semiconductor CoFe_2_O_4_ to the oxidized semiconductor WO_3_ [53]. At the same time, due to the transfer of electrons, the depletion of electrons in the reduced semiconductor and the accumulation of electrons in the oxidized semiconductor cause, respectively, upward and downward bending of the energy bands near the contact surfaces of CoFe_2_O_4_ and WO_3_ [54]. Under the combined effects of the spatial influence from such energy band bending, the mutual repulsion of the homogeneous charges by the Coulomb force, and the electron driven by the built-in electric field, the excited electrons can be transferred from the downward-bended CB of WO_3_ to the upward-bended VB of CoFe_2_O_4_ to combine with holes in the VB band of CoFe_2_O_4_, while the reverse transfer of electrons from the CB of CoFe_2_O_4_ to the CB of WO_3_ cannot occur [55]. The construction of this heterojunction is successful in allowing the WO_3_ to retain its high oxidizing activity at the expense of its lower reducing activity. Meanwhile, the composite material better spatially realizes the effective separation of photogenerated carriers [56]. The effectively separated photogenerated electrons react with O_2_ to generate superoxide radical (·O_2_^−^) near CB with a more negative potential, while the generated h^+^ react with H_2_O or OH^−^ to generate hydroxyl radical (·OH) near VB with a more positive potential. These generated radicals fully degrade the tetracycline pollutants.

## 3. Experimental Section

### 3.1. Synthesis of Materials

All the purchased reagents/chemicals for the experiment were of analytical grade and used as such without further refinement. Ferric nitrate ninhydrate, sodium hydroxide, and potassium persulfate were purchased from Sinopharm Chemical Reagent Co., Ltd. (Shanghai, China). Sodium tungstate and cobalt nitrate hexahydrate were purchased from Shanghai Mclean Biochemical Technology Co. Ethanol was purchased from Yunnan Yang Lin Industrial Development Zone, Shan Dian Pharmaceutical Co. Tetracycline was purchased from Shanghai Aladdin Biochemical Technology Co. N-butanol was purchased from the Luoyang City Chemical Reagent Factory. Sodium oxalate was purchased from Tianjin Hengxing Chemical Reagent Manufacturing Co. Ascorbic acid was purchased from Shanghai Titan Scientific Co (Shanghai, China). Nitric acid was purchased from Chongqing Chuandong Chemical Industry (Group) Co. (Chongqing, China), and deionized water (conductivity 18.25 μs/cm) was homemade in the laboratory.

WO_3_ preparation: For the preparation, 0.3299 g Na_2_WO_4_·2H_2_O was accurately weighed and dissolved in 15 mL of deionized water, and 10 mL of HNO_3_ (0.25 mol/L) was added to it with the help of a measuring cylinder. The mixture was stirred for 10 min and then transferred to a 100 mL sealed reactor for hydrothermal synthesis and heated for 12 h in an oven at 180 °C. The cooled mixture was then washed three times with deionized water and anhydrous ethanol. The prepared photocatalyst was dried at 60 °C. After grinding, it was put in a muffle furnace and calcined at 500 °C for 2 h (20 °C/min).

Preparation of CoFe_2_O_4_: For the preparation, 1.0812 g Fe(NO_3_)_3_·9H_2_O and 0.5826 g Co(NO_3_)_2_·6H_2_O were accurately weighed with an analytical balance and dissolved in 50 mL of deionized water under magnetic stirring until the solution was transparent. The pH of the mixture was regulated to 10 with NaOH (1M) solution. After stirring for 10 min, it was poured into a 100 mL sealed reactor for hydrothermal synthesis and heated for 10 h at 160 °C. After that, it was cooled down to room temperature and then washed three times each with water and ethanol. The dried photocatalysts were grinded and calcined in a muffle furnace at 600 °C for 3 h (10 °C/min temperature rise).

CoFe_2_O_4_/WO_3_ preparation: For the preparations, 2.13 mL, 4.3 mL, 6.4 mL, 8.5 mL, and 12.8 mL of Fe(NO_3_)_3_·9H_2_O (0.01 mol/L) solution were prepared separately and, respectively, mixed with 1.07 mL, 2.13 mL, 3.2 mL, 4.3 mL, and 6.4 mL solutions of Co(NO_3_)_2_·6H_2_O under stirring. After adding about 100 mg WO_3_ to each solution, the mixtures were heated for 10 h at 160 °C in a sealed reactor for hydrothermal synthesis. After that, each sample was cooled down to room temperature and then washed three times each with water and ethanol. The dried photocatalysts were grinded and calcined in a muffle furnace at 600 °C for 3 h (10 °C/min temperature rise).

### 3.2. Characterization

The crystal structures of photocatalysts were evaluated by means of an X-ray powder diffractometer (XRD, DX-2700BH, Dandong Haoyuan Instrument Co., Ltd., Liaoning, China) with a CuKα radiation source. The microstructure of samples was obtained by scanning electron microscopy (SEM, JSM 7600), and the elemental composition of the material was characterized by EDS. The light absorption characters were measured with the help of a UV-2600i UV-Vis spectrophotometer (UV-vis, Shimadzu, Suzhou, China) and an integrating sphere assembly. Fluorescence intensity was detected at the sample excitation wavelength by a fluorescence spectrophotometer model (Spectrofluorometer-FS5 (PL, Edinburgh Instruments Ltd., Edinburgh, UK). The specific surface area of the materials was analyzed using a fully automated high-performance physical adsorption tester (BET, BSP-PS4T).

### 3.3. Photocatalytic Activity

A 10 mg sample was weighed into a beaker and mixed with 50 mL of tetracycline solution (20 mg·L^−1^). Dark adsorption experiments were carried out after 2 min of ultrasonication (30 min to reach equilibrium between adsorption and desorption), and the absorbance of about 4 mL sample was measured after the saturation of adsorption.

Photocatalytic experiments were carried out with WO_3_, CoFe_2_O_4_, and CoFe_2_O_4_/WO_3_ composites with different loadings using a photocatalytic device with a Xenon lamp. Tetracycline was used as a model pollutant. Next, 10 mg of photocatalyst and 50 mL of tetracycline solution (20 mg/L) were added to a beaker and sonicated for 2 min. The mixture was kept under a Xenon lamp at a distance of 20 cm, and the experiments were carried out after the dark adsorption equilibrium was reached. Before the photocatalytic reaction was started, 15 µL of hydrogen peroxide was added to the system, and the photocatalytic experiment was extended to 20 min. Next, 4 mL of the reaction solution was taken at the end of the reaction to detect the absorbance in the wavelength range of 200–600 nm. The degradation rate of tetracycline was calculated by Equation (5):

(5) $$D\left(\%\right)=\frac{A_{0}-A}{A_{0}}=\frac{C_{0}-C}{C_{0}}\times 100\%$$

Here, D is the degradation efficiency, A_0_ and A are the absorbances, and C_0_ and C are, respectively, the concentrations of the stocked tetracycline solution and the solution irritated under light for a certain period.

To detect the active species majorly responsible for the removal of tetracycline, several different quenching agents such as ascorbic acid, potassium persulfate, n-butanol, and sodium oxalate were introduced to the photocatalytic system to trap, respectively, ·O_2_^−^, e^−^, ·OH, and h^+^. The photocatalytic reactions were conducted in the same manner as discussed for the measurement of the photocatalytic activity.

At the end of the photocatalytic experiments, the optimized photocatalyst with the best degradation efficiency was filtered and separated. The sample was dried at 80 °C in an oven and subjected to XRD inspection. The post-reaction XRD patterns of the samples were compared with the pre-reaction XRD patterns to evaluate the catalyst stability.

### 3.4. Photoelectrochemical Test

Ten mg of photocatalyst was dispersed in a mixture of 5 mL each of ethanol and ethylene glycol under sonication for 30 min followed by vigorous stirring for 4 h to form a homogenous suspension. Next, 20 µL of the formed suspension was evenly coated on ITO conductive glass, followed by natural air drying for 30 min. The open-circuit potential was measured in a 0.1 mol-L^−1^ Na_2_SO_4_ solution. To deeply investigate the separation efficiency of photogenerated e^−^-h^+^ pairs, the instantaneous photocurrent response curves (I-t) of the samples were obtained on the electrochemical workstation of the CHI-660D system using 0.1 of mol∙L^−1^ Na_2_SO_4_ as the electrolyte. Electrochemical impedance spectroscopy (EIS) was performed in a 0.1 mol-L^−1^ KCl solution containing 5 mmol·L^−1^ K_3_(Fe(CN)_6_]/K_4_(Fe(CN)_6_)(1:1).

## 4. Conclusions

In this study, WO_3_, CoFe_2_O_4_, and CoFe_2_O_4_/WO_3_ composite nanomaterials with different mass ratios were prepared by loading CoFe_2_O_4_ on the surface of WO_3_ using the in situ growth method. The prepared materials were characterized using XRD, SEM, and BET, which proved that the prepared materials were in accordance with the relevant study. The results analyzed by UV-Vis DRS, PL, EIS, and AC impedance showed that the photogenerated carriers produced by 5%CoFe_2_O_4_/WO_3_ subjected to excitation had longer lifetimes, and the composite had a stronger spectral response in the UV-visible region. From the experimental observations, it was concluded that in the case of sample dosages of 10 mg and 20 mg/L of tetracycline, the photoreaction of the 5%CoFe_2_O_4_/WO_3_ sample in 20 min had the highest degradation rate as the degradation efficiency reached 77%. This demonstrated that the introduction of CoFe_2_O_4_ enhanced the optical properties of WO_3_, and CoFe_2_O_4_/WO_3_ was an excellent photocatalyst. We believe that the higher photocatalytic performance of the composites is attributed to the S-scheme heterojunction, which allows the composites to achieve an efficient spatial separation of photogenerated electrons and holes without sacrificing stronger oxidizing properties.

## Figures and Tables

**Table 1:** Sample-specific surface area, pore diameter, and pore volume test results.

Sample	BET Specific Surface Area (m^2^/g)	Pore Size (nm)	Pore Volume (mL/g)
CoFe_2_O_4_	27.5202	52.1106	0.4280
WO_3_	10.3757	22.2659	0.0686
5%CoFe_2_O_4_/WO_3_	4.7766	20.0900	0.0308

## Data Availability

Data are contained within the article.
